# Three-Staged Surgical Strategy as a Combined Approach for Multilevel Cervical Pyogenic Spondylodiscitis

**DOI:** 10.7759/cureus.17747

**Published:** 2021-09-05

**Authors:** Manabu Mutoh, Toshiki Fukuoka, Osamu Suzuki, Shinnosuke Hattori

**Affiliations:** 1 Department of Neurosurgery, Nagoya University Graduate School of Medicine, Nagoya, JPN; 2 Department of Neurosurgery, Nagoya Ekisaikai Hospital, Nagoya, JPN

**Keywords:** cervical pyogenic spondylodiscitis, combined anterior posterior surgery, anterior cervical corpectomy, spinal instrumentation, halo brace

## Abstract

Cervical pyogenic spondylodiscitis is rare but can lead to severe clinical problems that often require aggressive surgical treatment for neurological deterioration and life-threatening conditions. Although combined surgical procedures are often utilized to treat multilevel cervical regions, there is a clinical debate regarding the appropriate order and timing of surgeries using the anterior and posterior approaches. Here, we report a case of severe multilevel cervical pyogenic spondylodiscitis treated using a three-staged surgical strategy consisting of cervical laminectomy, posterior fixation, and anterior corpectomy and fusion with an autologous long bone graft; the outcome was quite favorable. Our report demonstrates the safety and usefulness of three-staged surgery in the multilevel cervical region, especially under urgent situations.

## Introduction

Cervical pyogenic spondylodiscitis is rare; it can, however, cause serious clinical problems that often require aggressive surgical treatment for neurological deterioration and life-threatening conditions. The annual incidence of pyogenic spondylodiscitis is reported to be 0.5 to 5.78 cases per 100,000 individuals [1-4]. Regarding the site of pyogenic spondylodiscitis, the cervical spine is an uncommon site; it comprises approximately 4%-19% of all spinal spondylodiscitis cases. Moreover, the involvement of three or more vertebral levels at the cervical spine is more uncommon because its incidence is only 4.3%-13.3% of all cervical pyogenic spondylodiscitis cases [2,5-7].

Cervical pyogenic spondylodiscitis tends to exhibit rapid progression, leading to severe neurological deficits; hence, early surgical treatment is required in cases showing signs of structural instability or neurological deterioration [7-9]. However, there are few reports on the strategy of medical and surgical treatments for multilevel cervical pyogenic spondylodiscitis. Herein, we report a case of multilevel cervical pyogenic spondylodiscitis treated with a three-staged surgical strategy comprising laminectomy, posterior fixation, and anterior multilevel corpectomy and fusion with an autologous long bone graft; the outcome was quite favorable.

## Case presentation

**Figure 1:**
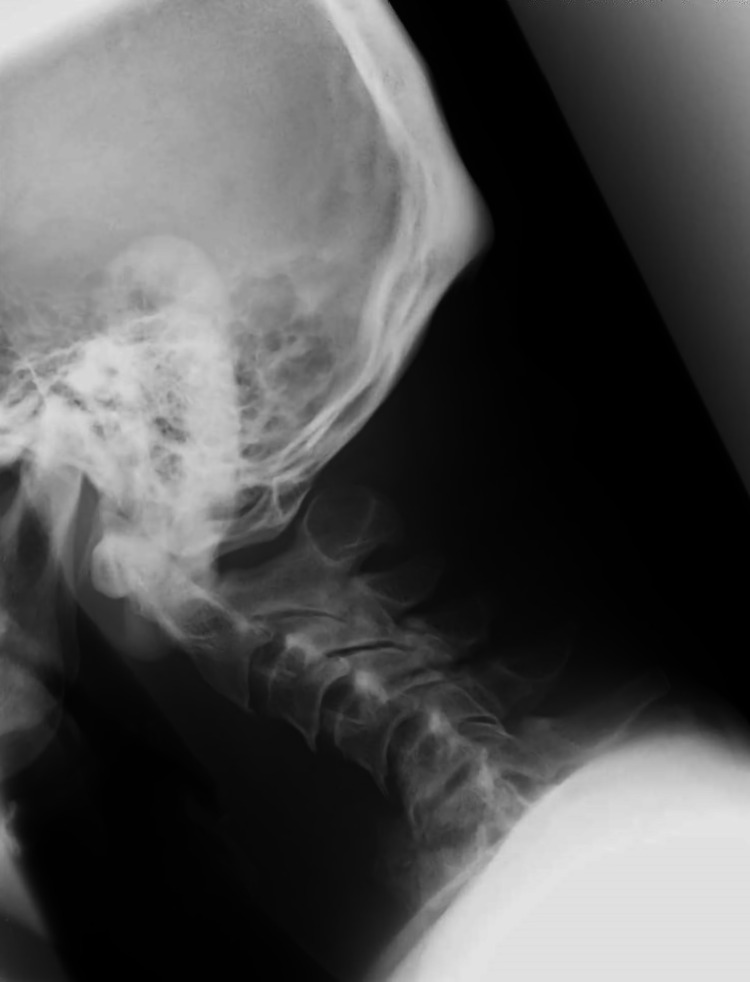
Preoperative cervical radiography Lateral view of the cervical radiograph on the admission day revealed severe destruction of the vertebral bodies and kyphotic changes at the C5–C7 vertebral levels.

**Figure 2:**
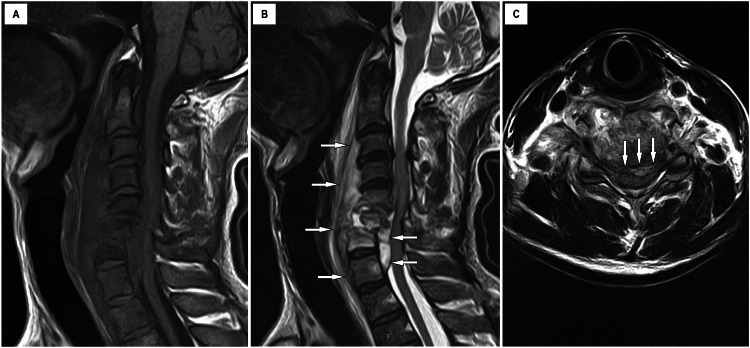
Preoperative cervical plain MRI (A) Sagittal view of cervical T1-weighted MRI revealed erosion and collapse of the C5, C6, and C7 vertebral bodies. (B) Sagittal view of cervical T2-weighted MRI. Arrows indicate epidural abscess from C3 to Th1. (C) Axial view of cervical T2-weighted MRI at the C6 level revealed severe spinal cord compression due to massive epidural abscess and collapsed vertebral bodies. Arrows indicate epidural abscess at the C6 level.

A 47-year-old man with a medical history of severe atopic dermatitis presented at the local doctor complaining of fever 37 days before consultation. Although the microorganism was identified as methicillin-sensitive *Staphylococcus aureus* in blood cultures, the origin of fever was unidentified at that time. Therefore, he was administered first-generation cephalosporin (unknown dosage) for two weeks and was confirmed negative blood culture test at the local hospital. After discharge from the local hospital, he had experienced numbness extended from both upper limbs to both lower limbs exacerbated by cervical flexion two days before consultation. He presented at our emergency department on Friday midnight complaining of neck pain, quadriplegia (American spinal injury association impairment scale Grade C), numbness of entire limbs, and bladder bowel dysfunction. Although he was afebrile, his laboratory data showed a remarkable inflammatory response; a white blood cell count and C-reactive protein level of 14,200/µL and 19.46 mg/dL, respectively. Cervical radiography revealed severe destruction of the vertebral bodies and kyphotic changes at the C5-C7 vertebral levels (Figure 1). Cervical plain magnetic resonance imaging (MRI) revealed a massive epidural abscess from C2 to Th1 and erosion and collapse of C5, C6, and C7 vertebral bodies, resulting in canal stenosis and spinal cord compression (Figures 2A-2C). He was diagnosed with severe cervical pyogenic spondylitis involving two intervertebral discs (C5/6 and C6/7) and three vertebral bodies (C5-C7). The patient was in immediate need of antibiotics and surgical treatment because the blood test indicated sepsis, and the severe neurological deficits rapidly progressed. The blood cultures were performed at our hospital followed by the administration of 2 g of cefazolin sodium three times per day. Initially, we considered the anterior approach as an option of surgical strategy; however, the present case required urgent surgery and was affected the multilevel vertebral bodies. Considering the difficulty and the high rate of surgical complications and fusion failures risk of one-stage surgical treatment using the anterior approach for multilevel vertebral bodies, we adopted a three-staged surgical strategy for immediate relief of the spinal cord compression and elective reconstruction of the anterior columns.

**Figure 3:**
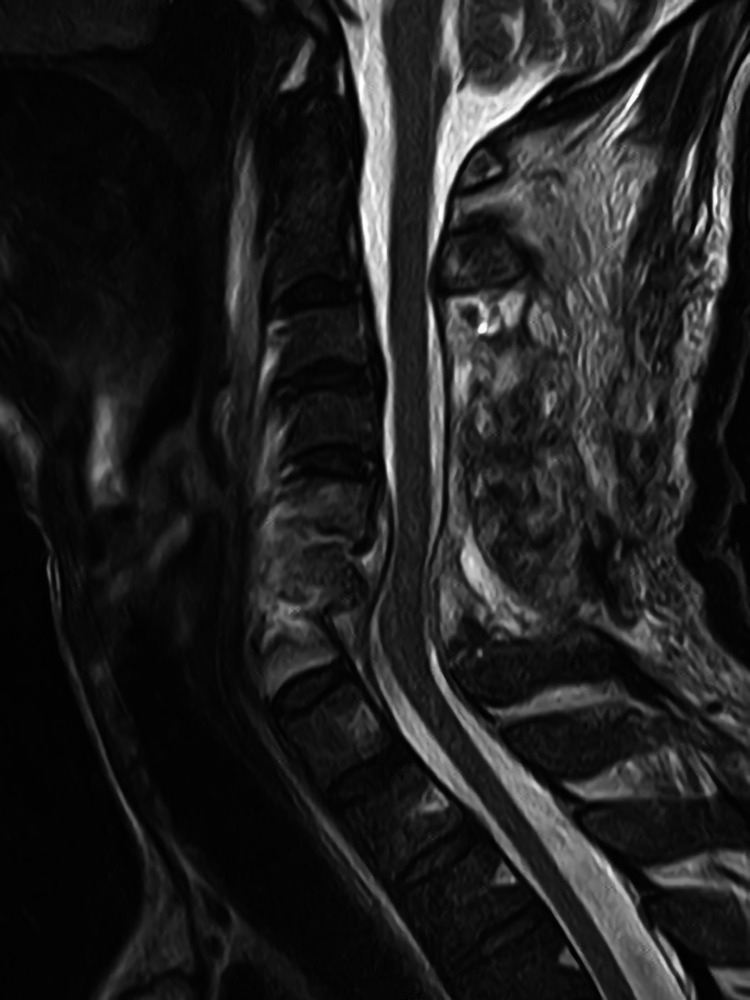
Cervical plain MRI after the first surgery Sagittal view of cervical T2-weighted MRI after laminectomy. Sufficient decompression of the spinal cord was achieved.

**Figure 4:**
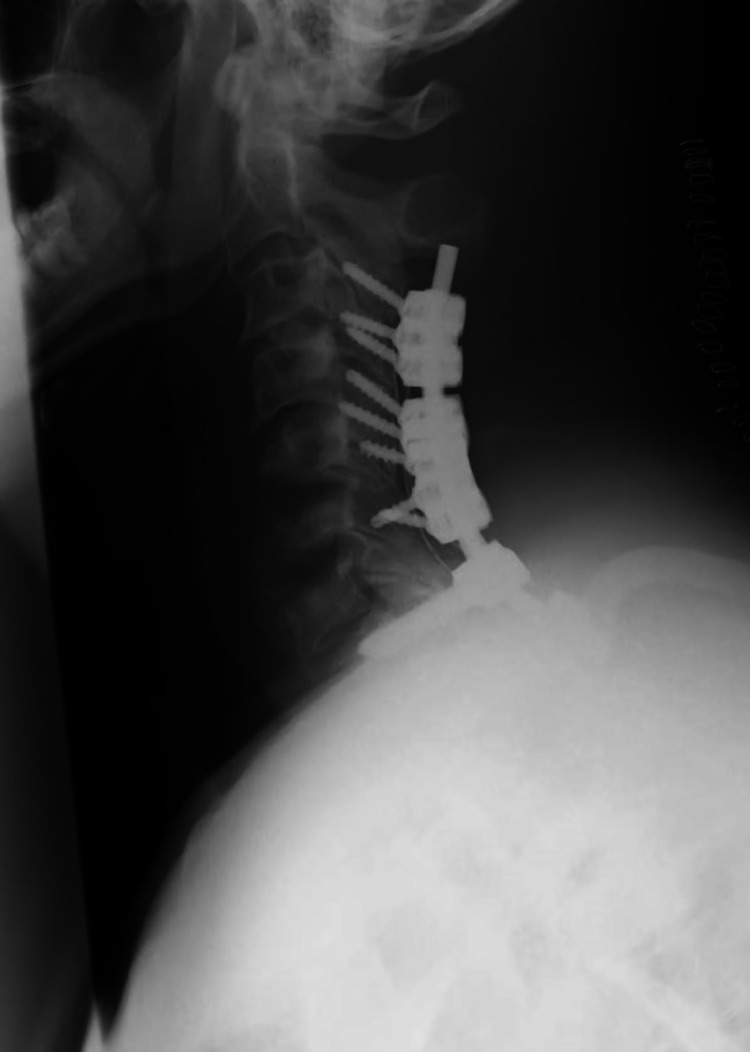
Cervical radiography after the second surgery Lateral view of the cervical radiograph after posterior fixation. Lateral mass screws were placed bilaterally at C3, C4, and C5 and on the left side at C6. A transarticular screw was placed on the right side at C6 due to the small lateral mass. Pedicle screws were placed bilaterally at Th1 and Th2. The pedicle screws at C7 were skipped because of pedicle fragility due to the spread of infection.

**Figure 5:**
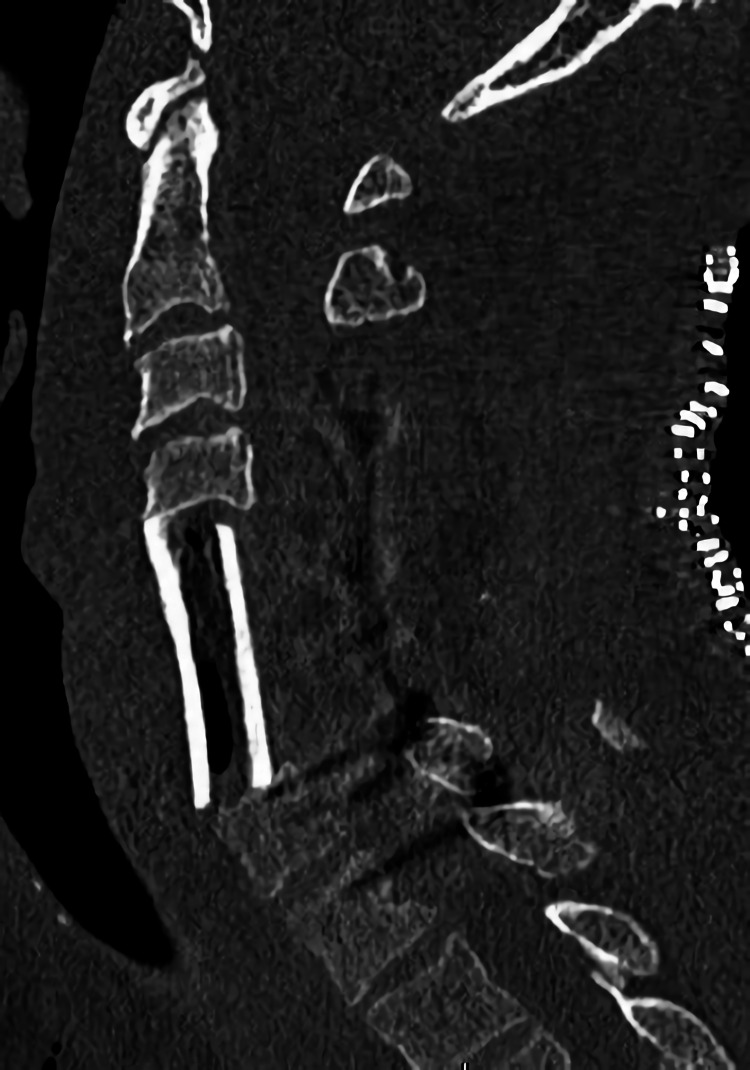
Cervical plain CT scan after the third surgery Sagittal view of the cervical CT scan after the third surgery of anterior corpectomy and fusion with an autologous long bone graft.

On the admission day (day 1), we performed emergency C3-C7 laminectomy and Th1 partial laminectomy for relieving spinal cord compression. Postoperative cervical MRI indicated sufficient spinal cord decompression (Figure 3). After the surgery and halo vest placement, ambulation and rehabilitation were started. On day 12, we performed the second surgery: posterior fixation from C3 to Th2. The dekyphosis of C5-C7 was successfully achieved using the intraoperative cervical position and posterior reduction with the screw instruments (Figure 4). On day 17, we performed the third surgery: anterior cervical debridement of the abscess, corpectomy of C5-C7 vertebral bodies, and reconstruction by transplantation of a 6-cm long autologous bone graft harvested from the left tibia, followed by placement of the halo vest again after the surgery (Figure 5). The infection-causing microorganism was identified as methicillin-sensitive *S. aureus* in blood cultures. Therefore, we continued intravenous administration of cefazolin sodium for six weeks, then switched to oral maintenance treatment of cefaclor until the erythrocyte sedimentation rate normalized. Although the obvious entry source of bacteria was unknown in the present case, he had a medical history of severe atopic dermatitis, which could be the entry source of *S. aureus*.

**Figure 6:**
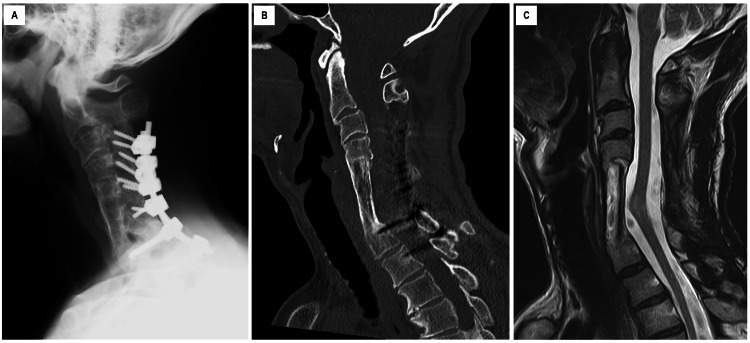
Cervical radiography, cervical plain CT scan, and plain T2-weighted MRI after two years and seven months of follow-up (A) Lateral view of the cervical radiograph. (B) Sagittal view of the cervical CT scan revealed that vertebral bodies from C4 to Th1 were favorably fused with a long autologous bone graft in a good cervical alignment. (C) Sagittal view of cervical T2-weighted MRI revealed sufficient decompression of the spinal cord.

After continuous antibiotic treatment and rehabilitation, he was discharged on day 109 with remarkable neurological improvement and could return to his original work. External fixation with the halo vest was continued for approximately three months until the transplanted bone graft was judged to have stabilized on radiography and computed tomography (CT) scan. No symptom or infection recurrence has occurred during the past two years and seven months of follow-up. The final follow-up cervical plain CT scan and MRI revealed that the vertebral bodies from C4 to Th1 were favorably fused with the bone graft, yielding good cervical alignment and sufficient spinal cord decompression (Figures 6A-6C). Informed consent was obtained from the patient for this case report.

## Discussion

Although multilevel cervical pyogenic spondylodiscitis is rare, surgeons are occasionally required to adopt a reliable medical and surgical treatment strategy under urgent situations. The anterior and posterior combined surgical procedure is often utilized for the treatment of multilevel cervical pyogenic spondylodiscitis since the anterior approach surgery alone has a high rate of surgical complications and fusion failures, especially in the treatment of multilevel regions [2,6,10]. However, the combined surgical procedure needs to clarify the clinical issues regarding the appropriate order and timing of the anterior and posterior approaches in surgery. Although disrupting the posterior elements is inevitable for the combined surgical procedure, performing salvage surgery for failures or surgical complications of the anterior approach alone tends to be difficult because of the complicated anatomy of the anterior cervical spine and the risk of laryngeal edema and hematoma, especially in the treatment of the multilevel vertebral bodies. In the present case, for the purpose of emergency decompression of the spinal cord and safe reconstruction of the anterior cervical columns, we created a three-staged surgical strategy consisting of cervical laminectomy, posterior fixation, and anterior corpectomy and fusion with an autologous long bone graft.

The three-staged surgical strategy might have the following three clinical advantages in cases such as the present case: First, decompression surgery using laminectomy is safe and easy to perform even in neurologically urgent situations, and it is not necessary to directly implant the instruments into the active infection site. Besides, the safety and efficacy of anterior cervical instrumentation for pyogenic spondylodiscitis are still undetermined [4,7,11]. Second, dividing the surgical treatments into several stages makes it easier to plan for the surgical method and the appropriate timing of the following surgery considering the patient’s perioperative condition. The more severe the case, the more difficult it is to predict the perioperative clinical course. Third, posterior fixation in advance can prevent dislocation of the transplantation bone after anterior surgery, and alignment correction can be easily performed. Especially, a multilevel cervical corpectomy is a challenging procedure because of the high incidence of surgical complications and fusion failures [12].

Although the staged surgical strategy offers excellent advantages, it has few disadvantages. First, debridement of anterior vertebral abscess and ventral epidural abscess cannot be performed directly in the first surgery. However, sufficient decompression of the spinal cord was successfully achieved, and bacterial infection was well controlled with antibiotic treatment in the present case. Even if infection control is difficult with antibiotic treatment alone, performing posterior decompression in advance makes it possible to have sufficient time to plan for radical treatment by the anterior approach. Second, staged surgery increases the number of surgeries and extends the length of hospital stay, which burdens the patient. In the staged surgical strategy, informed consent of its purpose and need is essential for maintaining the patient’s motivation to complete the entire treatment.

## Conclusions

Cervical pyogenic spondylodiscitis is a rare but serious clinical condition that can create urgent clinical situations that require aggressive and challenging surgical treatment. We report a case of multilevel cervical pyogenic spondylodiscitis treated using a three-staged surgical strategy with favorable outcomes and discuss the usefulness and benefits of staged surgery. It is important not to hesitate to adopt staged surgery in cases of severe multilevel cervical spondylodiscitis for safe and reliable treatment, especially under urgent situations.
